# Pneumococcal genotype 23B1 as a driver of increased 23B serotype carriage, penicillin non-susceptibility, and invasive disease in Belgium: a retrospective analysis

**DOI:** 10.1128/jcm.01696-24

**Published:** 2025-03-12

**Authors:** Lara Dierckx, Juan Pablo Rodriguez-Ruiz, Esra Ekinci, Liesbet van Heirstraeten, Laura Willen, Lize Cuypers, Philippe Beutels, Kirsten Maertens, Stefanie Desmet, Heidi Theeten, Surbhi Malhotra-Kumar

**Affiliations:** 1 Laboratory of Medical Microbiology, Vaccine and Infectious Disease Institute, University of Antwerp 198686 , Antwerp, Belgium; 2 Centre for the Evaluation of Vaccination, Vaccine and Infectious Disease Institute, University of Antwerp 198686 , Antwerp, Belgium; 3 Department of Laboratory Medicine, National Reference Centre for Invasive Pneumococci, University Hospitals Leuven 60182 , Leuven, Belgium; 4 Laboratory of Clinical Microbiology, Department of Microbiology, Immunology and Transplantation, KU Leuven 26657 , Leuven, Belgium; 5 Centre for Health Economics Research & Modelling Infectious Diseases (CHERMID), Vaccine and Infectious Disease Institute, University of Antwerp 198686 , Antwerp, Belgium; Johns Hopkins University, Baltimore, Maryland, USA

**Keywords:** *Streptococcus pneumoniae*, 23B1 genotype, carriage rate, invasive pneumococcal disease

## Abstract

*Streptococcus pneumoniae* serotype 23B, a non-vaccine serotype, has shown an increasing prevalence and penicillin non-susceptibility among carriage and invasive pneumococcal disease (IPD) isolates. Recently, a novel penicillin non-susceptible genotype has emerged, named 23B1. In the framework of the Belgian pneumococcal carriage study, we studied the prevalence of 23B_0_/23B1 among 586 23B strains (2016–2022) in 172 day care centers from 6- to 30-month-old children and among 130 pediatric 23B IPD isolates (2007–2021). Pneumococci were whole genome sequenced to determine the capsular polysaccharide genotype and sequence type (ST). Antimicrobial susceptibility testing determined penicillin and amoxicillin MICs, as well as resistance to co-trimoxazole and levofloxacin. 23B carriage was stable during 2016 – 2022 except in the 2020–2021 winter season when it increased. The proportion of genotype 23B1 compared to 23B_0_ decreased from 2016 – to 2022 but remained consistently higher than 23B_0_. In 2020–2021, an increase in the proportion of 23B1 was reflected in an overall increase in 23B carriage. All increases in 23B IPD cases were almost entirely driven by 23B1. The median penicillin MICs were significantly different for 23B_0_ (0.03 mg/L) and 23B1 (0.25 mg/L). In 2021, increased intermediate levofloxacin susceptibility was noted in 23B. 23B1-associated ST2372 was the most prevalent ST in carriage and IPD during 2013–2022. We show that an increase in 23B carriage among children was paralleled in pediatric IPD in Belgium, reiterating the utility of pneumococcal surveillance in the day care population. Serotype 23B is reported worldwide as an important pediatric non-PCV13 serotype with reduced penicillin susceptibility, with 23B1 as the presumed driver for the increased prevalence.

**IMPORTANCE:**

During the COVID-19 pandemic, the 23B serotype of *Streptococcus pneumoniae* has increased in prevalence in healthy carriage isolates from Belgian day care centers and pediatric (younger than 18 years of age) invasive pneumococcal disease (IPD) isolates. Additionally, an increase in penicillin non-susceptibility was also observed within this serotype. Recently, a genetic variant of 23B, named 23B1, was discovered, which is known to be related to decreased penicillin susceptibility. We showed that increases in 23B prevalence in healthy carriage and IPD cases always coincided with 23B1 expansions, leading to higher penicillin non-susceptibility rates. Increases in 23B in the day care population paralleled pediatric 23B IPD increases, indicating the vital role of day care monitoring of pneumococcal carriage. Countries should stay vigilant for prevalence increases in *S. pneumoniae* serotype 23B, given the decreased susceptibility to penicillin and co-trimoxazole of the 23B1 variant.

## INTRODUCTION

*Streptococcus pneumoniae* continues to be one of the main causes of respiratory infection and meningitis despite the introduction of pneumococcal conjugate vaccines (PCVs) mainly in childhood programs. These vaccines target the most invasive serotypes, whose incidence as causative pathogens has been critically reduced in children since their introduction. The widespread implementation of PCV7 in Europe was succeeded in 2010–2011 by new formulations, including more serotypes (PCV13 and PCV10), which were even more effective in reducing total invasive pneumococcal disease (IPD). However, this has subsequently led to a rise in colonization and IPD cases by the so-called non-vaccine types (NVTs). The incidence of IPD caused by NVTs, which are not covered by current and newer vaccine formulations like PCV20, has increased in Europe since 2012, representing a third of all cases in 2018, where NVT 23B made up 21% of these non-PCV20 cases in children <5 years (1). Recently, a genetic variant or genotype of 23B has been reported in the UK, 23B1. This genotype presents the same polysaccharide capsule as the original genetic variant 23B_0_ but presents a divergent capsular operon sequence in the 3′ end and belongs to a different genetic lineage (2). In the UK, the overall number of serotype 23B in carriage isolates and IPD cases, as well as the 23B1 proportion (relative to 23B_0_), continued to increase after PCV usage (2–5), and all 23B1 isolates obtained from children suffering from community-acquired pneumonia were penicillin non-susceptible (6).

Belgian pneumococcal vaccination policy has seen some unique changes over the years after the inclusion of PCV7 in the national vaccination program (2007–2010), free of charge and with a recommended status, resulting in coverage rates of over 90%. PCV7 was replaced by PCV13 from 2011 onward (2011–2015), although a switch was made to PCV10 (2015/2016–2018) in 2015 in the region of Flanders and in 2016 in Wallonia. The return to PCV13 came in 2019 after advice from the Belgian Superior Health Council (2019–2022), following the steep increase in 19A IPD cases (7, 8). Previous studies confirmed 23B as a dominating NVT in carriage during the PCV10 period in Belgium in healthy children (9). Despite high carriage rates, serotype 23B has been shown to have a low invasive disease potential (10). In the yearly reports from the Belgian National Reference Centre (NRC) for Invasive Pneumococci, the absolute number of 23B IPD cases in the <16 years age group has always been below 10 cases during the PCV7 period (2007–2010) and at the beginning of the PCV13 introduction (2011–2012) (*n* = 0–7, 0.0%–2.1% of all cases) (8). However, this changed from 2013 onward, 2 years after PCV13 introduction since the 23B cases rose in this age group and remained consistent at around 10 cases each year from 2013 to 2020 (*n* = 9–13, 3.9%–10.1% of all cases), although the 23B proportion increased from 3.9% in 2013 to 10.1% in 2020. In 2019, the year when PCV13 was re-introduced, NVT 23B was also mentioned as a predominating serotype in IPD isolates from children younger than 2 years and even became a top five serotype in pediatric IPD isolates in 2020, indicating its importance in this age group. In 2021–2022, the highest number of serotype 23B IPD cases in children <16 years old was reported in Belgium (20 cases in 2021 and 21 cases in 2022), but again a decrease to 13 23B cases in this age group was observed in 2023 (8). Among adult patients, the 23B serotype never made up more than 3.7% of all adult IPD cases, and this difference in age group prevalence was also reported in 2020 (8).

We investigated the prevalence of the 23B serotype and its genotypes in carriage strains from children aged 6 to 30 months attending Belgian day care centers (DCCs) during the PCV10 and second PCV13 period up until the winter season of 2021–2022, and in IPD strains from children younger than 18 years of age from 2007 to 2021, covering the different PCV periods in Belgium.

## MATERIALS AND METHODS

### Carriage isolates

In the framework of the Belgian pneumococcal carriage study, a total of 6,682 nasopharyngeal swabs were collected between 2016 and 2021–2022, mostly from October to May, in 172 DCCs in Belgium from children between 6 and 30 months old (11, 12). The child’s guardian completed questionnaires regarding the age, gender, preterm birth, breastfeeding, antibiotic use in the last 3 months, and the pneumococcal vaccination status of the child (Table S1). Rhinitis symptoms were recorded during sampling. Samples were collected in all provinces of Belgium, proportionally to the population density. Of these, an average of 65.7% (*n* = 3,713, minimum 58.1% and maximum 69.8% per season) tested positive in culture for *Streptococcus pneumoniae*. Only pneumococcal isolates that were serotyped as 23B (Quellung reaction) were included in the present study (*n* = 586) (Table 1).

**TABLE 1:** Number of pneumococcal serotype 23B isolates for each season from 2016 to 2021–2022 from children attending DCCs (6–30 months old) and from IPD patients younger than 18 years old from 2007 to 2021 in Belgium, divided into 23B_0_/23B1 isolates with their respective proportion in 23B isolates

Vaccination	Year^*a*^	Pneumococcal isolates (*n*)	23B isolates and prevalence (*n*, %)	23B_0_ proportion among 23B (*n*, %)	23B1 proportion among 23B (*n*, %)
Pneumococcal isolates from children attending day care centers					
PCV13/PCV10	2016	462	61 (13)	15 (25)	46 (75)
PCV10	2016–2017	748	124 (17)	30 (24)	94 (76)
PCV10	2017–2018	665	91 (14)	32 (35)	59 (65)
PCV10	2018–2019	484	72 (15)	27 (38)	45 (62)
PCV13	2019–2020	478	68 (14)	29 (43)	39 (57)
PCV13	2020–2021	467	102 (22)	22 (22)	80 (78)
PCV13	2021–2022	409	68 (17)	27 (40)	41 (60)
	All seasons	3,713	586 (16)	182 (31)	404 (69)
Pneumococcal isolates from IPD patients younger than 18 years old					
PCV7	2007	354	2 (0.6)	2 (100)	0 (0)
PCV7	2008	355	4 (1.1)	3 (75)	1 (25)
PCV7	2009	443	5 (1.1)	5 (100)	0 (0)
PCV7	2010	386	0 (0)	/^*b*^	/
PCV13	2011	446	7 (1.6)	5 (71)	2 (29)
PCV13	2012	282	6 (2.1)	4 (67)	2 (33)
PCV13	2013	258	10 (3.9)	3 (30)	7 (70)
PCV13	2014	172	10 (5.8)	0 (0)	10 (100)
PCV13/PCV10	2015	180	8 (4.4)	3 (38)	5 (62)
PCV13/PCV10	2016	171	10 (5.8)	1 (10)	9 (90)
PCV10	2017	203	13 (6.4)	5 (38)	8 (62)
PCV10	2018	215	10 (4.7)	4 (40)	6 (60)
PCV10/PCV13	2019	225	13 (5.8)	3 (23)	10 (77)
PCV13	2020	129	12 (9.3)	3 (25)	9 (75)
PCV13	2021	163	20 (12)	2 (10)	18 (90)
	All years	3,982	130 (3.3)	43 (33)	87 (67)

^*a*^ Samples were collected during the winter season.

^*b*^ ‘/’ denotes no genotype data.

### Invasive isolates

All isolates (*n* = 130), identified as serotype 23B by the Quellung test, from pediatric IPD cases in children younger than 18 years old from all over Belgium, received at the NRC during the period 2007 to 2021, were included in this study. All IPD isolates were obtained from blood, cerebrospinal fluid, pleural fluid, or joint fluid obtained from patients presenting with meningitis, bacteremia, pneumonia, or sepsis (Table S1). Details of the surveillance at the NRC have been previously described (7). Age, gender, location, and the clinical diagnosis of the patient were also registered. The IPD samples used in the present study covered a wider period than the carriage samples and also included the period when PCV7 (2007–2010) and PCV13 (2011–2015) were in use in Belgium.

### Whole-genome sequencing

We performed whole-genome sequencing to differentiate 23B1 from 23B_0_ on all isolates described above (*n* = 716). Multiple pneumococcal colonies from a pure culture were inoculated into Todd-Hewitt broth (BD Bioscience) and left to grow overnight in a 5% CO_2_ incubator at 37°C. DNA was extracted using the MasterPure Complete DNA and RNA Purification Kit (LGC Biosearch Technologies, Hoddesdon, UK), and a further purification step was performed using the genomic DNA Clean & Concentrator-10 kit (Zymo Research). DNA concentration was determined with a Qubit3 Fluorimeter using the Qubit dsDNA HS Assay Kit (Invitrogen). Following this, the sequencing library was prepared, using the Nextera XT DNA Sample Preparation Kit (Illumina), and sequencing was done using the MiSeq reagent kit version 2 (Illumina).

The quality of the obtained reads was assessed with FastQC version 0.11.9. BacPipe was used for read trimming, sequence type (ST) assignment, antimicrobial resistance gene detection, genome assembly, and protein annotation (13). PneumoCaT was used to differentiate the capsular genotype from the raw reads (14). The assemblies underwent core genome alignment with Parsnp version 1.5.3 (15), using default settings and ATCC700669 as a reference. A single nucleotide polymorphism (SNP) distance matrix was obtained from the core genome alignment using snp-dists version 0.7.0 (https://github.com/tseemann/snp-dists). Phylogeny was inferred from the core genome alignment with RAxML version 8.2.12 using the GTRCAT option and 100 bootstraps (16), and the tree was visualized and annotated with iTOL version 7.0. Finally, PopPUNK version 2.4.0 was used to assign isolates to the Global Pneumococcal Sequencing project Clusters (GPSCs) (https://www.pneumogen.net).

### Minimal inhibitory concentration testing

The manual microbroth dilution method was used to determine MICs to penicillin and amoxicillin, and the results were interpreted according to EUCAST non-meningitis breakpoints (version 14.0, 2024). Isolates were considered penicillin non-susceptible when the penicillin MIC was >0.06 mg/L and amoxicillin non-susceptible when the amoxicillin MIC was >0.5 mg/L. Additionally, all cultured strains were tested at the NRC for a range of antibiotics (co-trimoxazole, erythromycin, tetracycline, and levofloxacin) with the disk diffusion method (Table S2).

### Statistics

The Chi-square (χ^2^) test was used to assess significant changes in carriage and IPD rates of 23B, 23B_0_, 23B1, and penicillin non-susceptibility in isolates depending on the clinical variables. Univariate testing was done to assess the difference in demographics. The Mann-Whitney *U*-test was used to assess the difference in median penicillin MIC between the 23B_0_ and 23B1 isolates.

## RESULTS

### Dominating 23B1 leads to increased penicillin non-susceptibility in serotype 23B pneumococci in carriage

**Fig 1:**
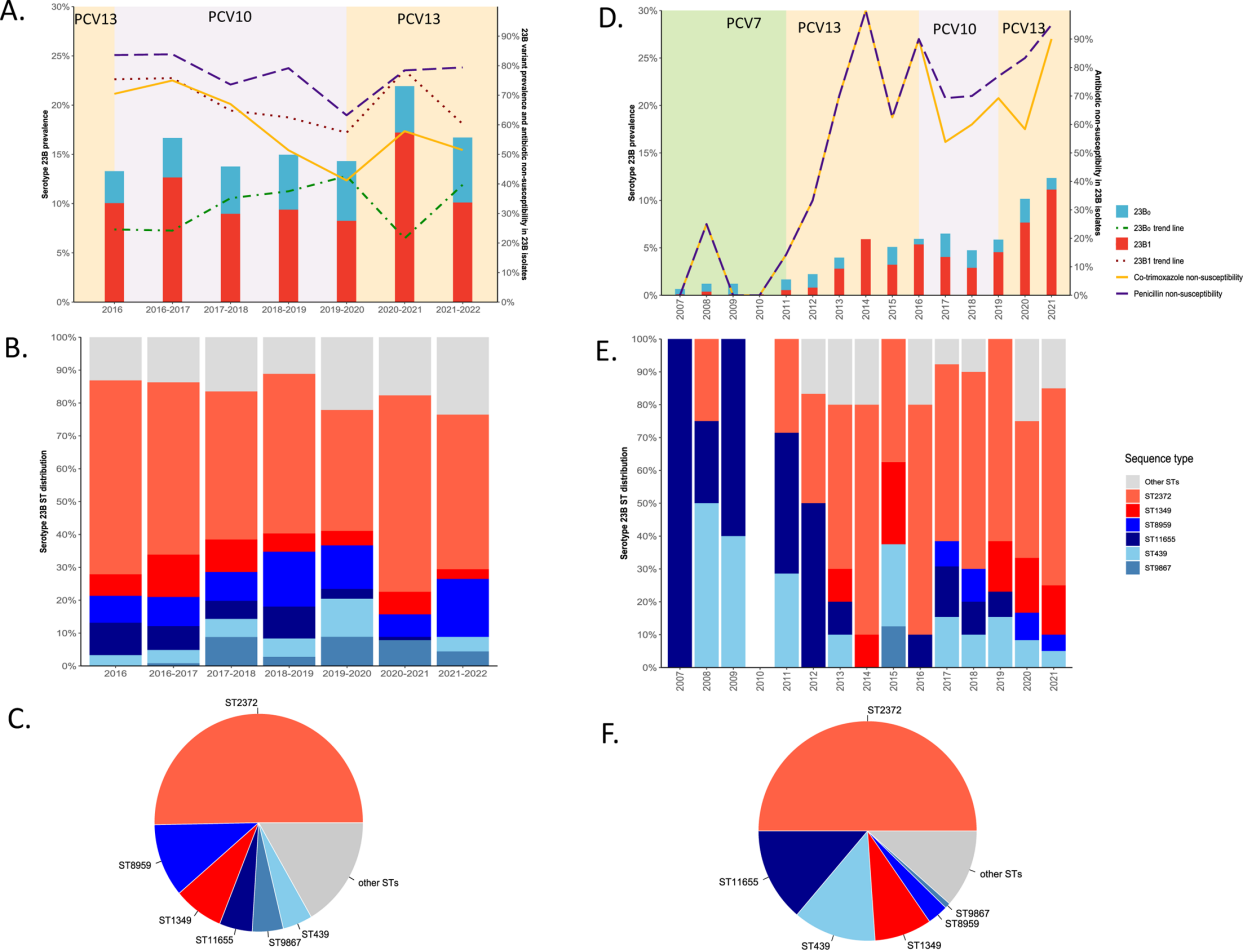
(A) Bar plot showing the 23B prevalence among carriage isolates from children attending DCCs (6–30 months old) in Belgium from 2016 to 2022 and colored according to 23B_0_/23B1 proportion, which is also depicted as a trend line. Penicillin and co-trimoxazole non-susceptibility in 23B isolates in each season is shown as a line chart. (**B**) Overview of the most prevalent STs in 23B isolates from children attending DCCs (6–30 months old) in Belgium from 2016 to 2022. (**C**) ST distribution of the 23B carriage isolates from children attending DCCs (6–30 months old) in Belgium. (**D**) Bar plot showing the 23B prevalence of IPD isolates from children (<18 years old) in Belgium from 2007 to 2021 and colored according to 23B_0_/23B1 proportion. Penicillin and co-trimoxazole non-susceptibility in 23B isolates in each season is shown as a line chart. (**E**) Overview of the most prevalent STs in 23B isolates from children (<18 years old) suffering from IPD in Belgium from 2007 to 2021. (**F**) ST distribution of the 23B IPD isolates from children (<18 years old) in Belgium.

Carriage of 23B isolates was stable during 2016–2022 (Fig. 1A, 13.2%–16.6%, *P* = 0.47), except in the 2020–2021 winter season when it increased significantly to 21.8% (*P* = 0.003). The proportion of genotype 23B1 relative to 23B_0_ significantly decreased from 2016 to 2022 (75.4%–60.3%, *P* = 0.009). In 2020–2021, an increase in the proportion of 23B1 was observed compared to the years before (78.4%, *n* = 80/102, *P* = 0.006), which overlapped with the aforementioned peak in overall 23B carriage.

Penicillin and co-trimoxazole non-susceptibility in all 23B isolates were high in 2016 (83.6%, *n* = 51/61 and 70.5%, *n* = 43/61) and slowly but significantly decreased until 2019–2020 (63.2%, *n* = 43/68, *P* = 0.01 and 41.2%, *n* = 28/68, *P <* 0.001). Afterward, penicillin non-susceptibility increased significantly in 2020–2021, proportionally following the dynamics of the 23B1 genotype (Fig. 1A). All isolates were susceptible to amoxicillin. Median penicillin MICs were significantly different for 23B_0_ (*n*_penicillin non-susceptible_ = 66/182) and 23B1 isolates (*n*_penicillin non-susceptible_ = 390/404), respectively, highlighting the primarily penicillin non-susceptible phenotype in 23B1 isolates. There were no significant differences between 23B_0_ and 23B1 isolates regarding other antibiotic susceptibilities (Table S2). However, there was an increase noted in levofloxacin intermediate susceptibility in the 23B isolates, unrelated to a variant, in 2020–2021 and even more in 2021–2022, where all 23B isolates were intermediate susceptible (0/68 [0.0%] to 46/102 [45.1%] to 68/68 [100%]).

**Fig 2:**
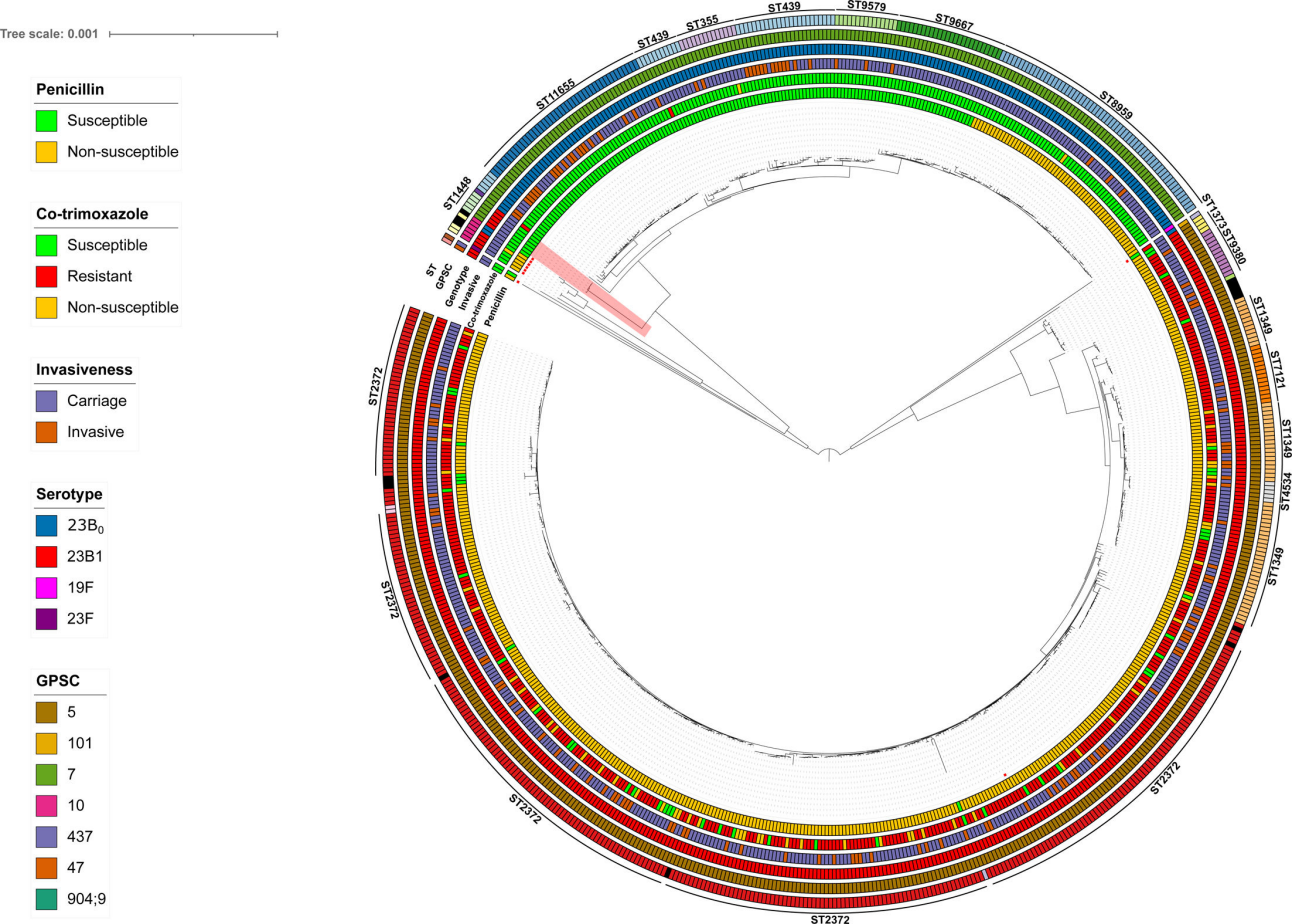
Phylogenetic tree generated from the study isolate assemblies. Inner red squares represent the presence of *erm(b)* and *tet(M)* resistance determinants, which correlate to erythromycin and tetracycline resistance, respectively. Penicillin and co-trimoxazole susceptibility is depicted in the inner rings. The third ring shows whether the isolate is invasive or is carried in the nasopharynx. Outer circles represent the serotype of the isolate, GPSC clustering, and ST (only STs presenting more than five isolates are shown with text, and novel STs are depicted in black). Finally, the red area indicates ST1448 23B1 isolates clustering with 23B_0_ isolates.

The most prevalent ST observed within the 23B_0_ population was the penicillin non-susceptible ST8959 (35.2%, *n* = 64/182), closely followed by penicillin-susceptible ST11655 (16.5%, *n* = 30/182) and ST9867 (15.4%, *n* = 28/182). In the 23B1 population, the most prevalent STs were ST2372 (73.0%, *n* = 295/404) and ST1349 (11.1%, *n* = 45/404), both being penicillin non-susceptible. Furthermore, ST2372 was the most common ST represented in our entire data set and was responsible for the peak 23B isolates in 2020–2021. Interestingly, none of the STs were shared between the 23B_0_ and the 23B1 groups (Fig. 1B, C, E, F, and 2).

There was no significant difference between 23B_0_ and 23B1 prevalence regarding the geographical distribution of DCCs in Belgium, gender, preterm birth, breastfeeding, rhinitis symptoms, and pneumococcal vaccination status. However, the mean age of children harboring 23B1 (19.7 months) was higher than those harboring 23B_0_ (18.4 months, *P* = 0.04). Interestingly, reported antibiotic use in the last 3 months did not influence the isolation of penicillin non-susceptible isolates, with similar rates of susceptible (24.8%, 30/121) and non-susceptible (24.1%, 105/435) isolates obtained from previously treated subjects (Table S1).

### 23B1 drove the increase in pediatric IPD caused by 23B in Belgium in 2021

During the PCV7 period (2007–2010), the number of pediatric IPD caused by serotype 23B in children younger than 18 years old was low and stable (Table 1; Fig. 1D, *P* = 0.10). After 2011, the prevalence of this serotype among children increased until peaking in 2020–2021 (Fig. 1D). This peak in prevalence of 23B in IPD coincided with the peak observed in 2020–2021 in the carriage isolates (Fig. 1A and D).

The 23B IPD cases found during PCV7 period were primarily of the 23B_0_ genotype (83.3%, *n* = 15/18). 23B1, although already found to cause IPD in 2008, only increased during the first vaccination period with PCV13, when 90% of 23B isolates (*n* = 9/10) were identified as 23B1. From 2019 onward, after the re-implementation of PCV13, the increase in serotype 23B among IPD cases was almost entirely driven by the increase in the 23B1 genotype All increases in 23B IPD cases from 2013 onward were associated with an increase in the 23B1 proportion, which was also associated with peaks in penicillin and co-trimoxazole non-susceptibility (Fig. 1D). All penicillin non-susceptible isolates were either 23B1 isolates or ST8959 23B_0_ isolates, but all isolates remained susceptible to amoxicillin. Of note, the 23B1 proportion and the co-trimoxazole non-susceptibility in 23B isolates in this data set were strongly associated with a correlation coefficient of 0.99 (*P* < 0.001). There were no significant differences between 23B_0_ and 23B1 isolates regarding other antibiotic susceptibilities (Table S2). However, levofloxacin intermediate susceptibility did spike in 2021 in these 23B invasive isolates compared to the years before (9/13 in 2019 to 3/12 in 2020 to 20/20 in 2021), which was also reflected in the carriage data of 2021–2022.

The most prevalent ST after 2013 was ST2372 (56.6%, *n* = 60/106), which was also the ST found in all five 23B1 isolates before 2013 (20.8%, *n* = 5/24) and was responsible for the 2021 peak (Fig. 1E).

Differences in geographic location, gender, age category, clinical diagnosis, and sample type between children with 23B_0_ and 23B1 isolates were not significant.

### 23B1 is genetically distinct from 23B_0_

23B_0_ and 23B1 isolates form two genomic distinct clades, with 11,670 core-genome SNPs (Fig. 2). Their genetic difference is also reflected in their lack of shared STs or GPSCs, with GPSC7 being dominant for 23B_0_ and GPSC5 for 23B1.

Within the 23B_0_ cluster, isolates presented an average of 1,957 core-genome SNPs, and within the 23B1 cluster, the number of core-genome SNPs decreased to 1,722, further demonstrating the within-genotype homogeneity and the large genomic distance between genotypes. Of interest are five ST1448 23B1 isolates, which are more related to the 23B_0_ isolates (4,991 core-genome SNPs) compared to the other 23B1 isolates (11,432 core-genome SNPs). Three 23B1 STs (ST386, ST1800, and ST4253) and two 23B_0_ STs (ST1078 and ST4677) showing divergent GPSCs were also distinct from the main two clades, although their occurrence was very rare in the data set (*n* = 6) (Fig. 2). Carriage of the resistance determinants *ermB* and *tetM* was common in these rare isolates.

## DISCUSSION

Our data highlight the importance of genotyping to understand changes in phenotypes, i.e., increase in antibiotic resistance, linked to increased proportions of specific serotype variants. We also show that the increase in 23B carriage among day care children went in parallel with an increase among IPD in Belgium, reiterating the utility of closely monitoring pneumococcal carriage in the day care population, as was also previously shown for 19A (17).

### Impact of PCV switches and COVID-19 pandemic

In this population, we do not observe an influence of previous antibiotic treatment on the selection of genotype 23B1 or non-susceptible isolates, although this non-susceptibility is short-lived (18). Considering this, together with the lack of amoxicillin non-susceptible isolates in this data set, antibiotic treatment is not the main driver, leaving vaccination and non-pharmaceutical interventions as the potential main drivers of the 23B1 expansion. However, the prevalence of penicillin non-susceptibility in this genotype calls for continued monitoring of the appearance of amoxicillin non-susceptibility, as the resistance mechanism is shared.

The IPD results confirmed that the 23B1 genotype has been in the population as early as 2008, in line with findings from other countries. The 23B1 genotype was reported in IPD in several countries (the UK, USA, and Thailand) already during 2005–2008, although the genotype’s prevalence remained low during this period, as observed in Belgium (2, 5). Subsequently, during 2016/2015–2019, which coincided with the PCV10 vaccination period in Belgium, an increased prevalence of 23B1 was observed. This prevalence doubled in 2021 during the COVID-19 pandemic and the second PCV13 implementation in Belgium. Although this genotype distribution could not be analyzed for the years 2022 and 2023, the Belgian NRC report shows a decline in the proportion of 23B serotype in IPD in children <16 years old (−2.9%) in 2022 and even further in 2023 (−3.9%) (8). During the PCV10 vaccination period in Belgium, serotype 23B was the most prevalent colonizer in carriage in both healthy infants in DCCs and in children with otitis media (9). However, in 2020–2021, 1 year after the re-implementation of PCV13, the overall 23B carriage and involvement in IPD, as well as the 23B1 proportion, peaked before decreasing again in 2021–2022. The loss of competition from PCV13 non-PCV10 serotypes (e.g., 19A) or natural variation after the switch to PCV13 may have resulted in an increase in overall 23B carriage due to increased 23B1 proportion. The increases also coincide with the start of the COVID-19 pandemic (2020), and the decrease in viral respiratory circulation because of the COVID-19 protective measures could have played a role. During the first 2 years of the COVID-19 pandemic, the Invasive Respiratory Infection Surveillance consortium reported a global decrease in IPD (19), although no change in pneumococcal serotype distribution was found (20). At the end of 2021, increases in IPD were reported following the loosening of stringent COVID-19 containment measures worldwide and the return of viral respiratory infections (20). In Belgium, the DCCs were never shut down as they were deemed essential services, which might have resulted in the continuation of the serotype distribution compared to the years before the COVID-19 pandemic. During season 2019–2020, the DCCs remained open with a 50% occupancy restriction. One season later, during 2020–2021, the occupancy was comparable to the seasons before the COVID-19 pandemic (E. Van den Bosch and E. Ekinci, unpublished data). However, other guidelines for the general public were in place regarding mask use for adults and social distancing, which in turn affected the rules and social contacts in the DCCs. From February 2022 onward in Belgium, the mask mandate and social distancing rules were relaxed for caretakers in DCCs and primary schools, possibly increasing viral transmission again within and between families (21) and co-occurred with a slight decrease in 23B carriage. However, a recent paper using the same DCC carriage samples found SARS-CoV-2 transmission to be very infrequent in this population during the COVID-19 pandemic from 2020 to February 2022, making the interaction between SARS-CoV-2 and pneumococcal carriage rate unlikely in this period of time (22). Nevertheless, the number of 23B IPD cases was too low to draw strong conclusions on its population dynamics and the effect of pharmaceutical and non-pharmaceutical interventions.

The co-occurring peaks in 23B carriage in healthy children and 23B IPD cases highlight the relevance of pneumococcal serotype monitoring in DCCs. Previously, the resurgence of 19A IPD after PCV10 introduction was also reflected in the DCC’s surveillance in Belgium and eventually led to the switch back to the 13-valent vaccine (8, 17). A German study also identified important PCV13 serotypes like serotypes 3 and 19A circulating in healthy carriage and matched it to IPD increases post-PCV10 in 2009 (23). Greece reported a significant 23B increase post-PCV10 and PCV13 introduction (respectively, May 2009 and June 2010) in children attending DCCs (24). In the years that followed, the Greek National Meningitis Reference Laboratory found 23B as the third most prevalent serotype found in pneumococcal meningitis cases, indicating the predicting value of a DCC surveillance study (25). Many countries use pneumococcal carriage monitoring to anticipate pneumococcal serotype trends in IPD post-PCV, as carriage surveillance was proven to be a reliable simple predictor (26, 27).

### Serotype 23B on the rise worldwide

Serotype 23B has been indicated worldwide as a serotype to watch in asymptomatic carriage and IPD, mostly in young children (Table S3). SpIDnet, a European IPD surveillance network, identified 23B as a dominating NVT in countries using specifically PCV13 (28). However, given that both genetic variants differ significantly with 11,670 core-genome SNPs as evident from Fig. 2 and was noted previously in the original UK study (2), it is interesting to speculate if these expansions were due to 23B1, as was the case in Belgium and the UK with 23B1-ST2372.

Studies from Sweden and Portugal described a rise in 23B prevalence in penicillin non-susceptible isolates from adult IPD samples in the PCV13 era (29, 30). As seen in the Belgian data, penicillin non-susceptible 23B isolates were mostly 23B1, suggesting an expansion of 23B1 rather than 23B_0_ in countries with rising penicillin non-susceptibility in 23B isolates. A Spanish clinical surveillance study identified 23B as a rising serotype from 2020 onward and related this with a rise in penicillin non-susceptibility in pneumococcal isolates (31).

Another Spanish study reported an increase in ST2372 23B isolates from pediatric IPD samples from 2011 onward, while in Norway, the rise in antimicrobial resistance in NVTs was also associated with an increase of specifically ST2372 23B IPD isolates, indicating an overall rise in ST2372 across Europe (32, 33). Another study from the USA, 3 years after PCV13 introduction, noted a 23B increase in IPD samples from children, more specifically, an increase in penicillin non-susceptible ST1373 23B isolates (34). Germany reported ST439 and ST1349 as the most common 23B STs in IPD samples after PCV13 implementation, causing a significant rise in 23B IPD (35). All these studies specifically mention a rise in ST2372, ST1373, or ST1349 23B isolates, which were STs exclusively found in 23B1 isolates in Belgium, making it likely that these increases are due to genotype 23B1. Moreover, in a UK carriage study in children, the 23B1 genotype completely overtook the 23B population, as 23B1 was the only variant found in all 23B isolates in 2018, after 8 years of continued PCV13 use (5). Iceland, however, implemented the PCV10 vaccine in 2011 and reported a significant increase in 23B carriage (from *n* = 1, 0.7/1,000 samples to *n* = 152, 49.3/1,000 samples; *P* < 0.001) in 2012–2017 compared to 2009–2011, specifically due to ST439, which is associated with 23B_0_ in our study, whereas 23B was found only once in the 3-year surveillance before PCV10 (36). Other countries implementing PCV10 as the first PCV in an infant program, like Finland, Bulgaria, and Brazil, did not report a rise in serotype 23B infections, whereas all these countries reported 19A as a common non-PCV10 serotype in the population, which is also reported by the European SpIDnet study (28, 37–39). At the same time, most of the aforementioned countries using PCV13 also reported a significant decrease in 19A carriage and an increase in suspected 23B1 cases. Therefore, countries using PCV13, contrary to countries with PCV10 usage, should be on guard for potential rises in IPD caused by NVT 23B, driven by genotype 23B1. This concern over 23B IPD infections was also the incentive to include serotype 23B in a new 21-valent PCV, stating that the newly included serotypes contribute substantially to the adult IPD burden (40).

### Conclusion

Peaks in 23B prevalence in 2021, during the second PCV13 implementation in Belgium and the COVID-19 pandemic, in both carriage and IPD samples coincide with a rise in the proportion of type 23B1 and penicillin non-susceptibility. In European and international studies, non-vaccine type 23B was indicated to be an important pediatric serotype, and expansions within this serotype are likely to be driven by the 23B1 genotype based on ST analysis.

## Data Availability

The data sets generated and analyzed during the current study are available at ENA under BioProject number PRJEB78821 and at NCBI with BioProject number PRJNA1144854.

## SUPPLEMENTAL MATERIAL

Table S1 jcm.01696-24-s0001.xlsx Overview of all the metadata and results of each 23B strain.

Tables S2 and S3 jcm.01696-24-s0002.docx Antibiotic non-susceptibility rates for the 23B carriage and IPD isolates, and overview of literature describing European and worldwide 23B increases in the population.

ASM does not own the copyrights to Supplemental Material that may be linked to, or accessed through, an article. The authors have granted ASM a non-exclusive, world-wide license to publish the Supplemental Material files. Please contact the corresponding author directly for reuse.
