# Supplementary material for: Pneumococcal genotype 23B1 as a driver of increased 23B serotype carriage, penicillin non-susceptibility, and invasive disease in Belgium: a retrospective analysis
Source: J Clin Microbiol. 2025 Mar 12;63(4):e01696-24. doi: 10.1128/jcm.01696-24 (PMC11980397; doi:10.1128/jcm.01696-24)
Supplement: Tables S2 and S3 — Antibiotic non-susceptibility rates for the 23B carriage and IPD isolates, and overview of literature describing European and worldwide 23B increases in the population. [file jcm.01696-24-s0002.docx]

**Supplementary table 1.** Excel file containing all of the results and metadata of the 23B carriage and IPD isolates which were sequenced in this study.

**Supplementary table 2.** Antibiotic non-susceptibility rates for the 23B carriage and IPD isolates. The susceptibility was tested using the disk diffusion method at the NRC.

| **Antibiotic** | **23B* (n=716)** | **23B0 (n=225)** | **23B1 (n=491)** | **Carriage isolates (n=586)** | **Invasive isolates (n=130)** |
| --- | --- | --- | --- | --- | --- |
| Erythromycin non-susceptibility | 27 (3.8%) | 11 (4.9%) | 16 (3.3%) | 27 (4.6%) | 0 (0.0%) |
| Levofloxacin non-susceptibility | 194 (27.1%) | 57 (25.3%) | 136 (27.7%) | 114 (19.5%) | 80 (61.5%) |
| Cefotaxime non-susceptibility | 1 (0.0%) | 0 (0.0%) | 1 (0.0%) | - | 1 (0.8%) |
| Tetracycline non-susceptibility | 25 (3.5%) | 11 (4.9%) | 14 (2.9%) | 25 (4.3%) | 0 (0.0%) |
| Co-trimoxazole non-susceptibility | 438 (61.2%) | 6 (2.7%) | 432 (88.0%) | 356 (60.8%) | 82 (63.1%) |
| *Resistant and intermediate susceptible were interpreted as non-susceptible isolates | | | |  |  |

**Supplementary table 3**. Overview of international papers describing the 23B prevalence and characteristics after nationwide vaccination campaigns. Red and blue words respectively indicate key 23B1 and 23B_0_ related characteristics seen in the Belgian population study.

| **Reference** | **Country** | **Period** | **PCV in study period (introduction year)** | **IPD/carriage** | **Age group** | **Serotype change** | **23B antibiotic susceptibility** | **23B ST mentioned** |
| --- | --- | --- | --- | --- | --- | --- | --- | --- |
| [29] | Sweden | 2016-2018 | PCV10 (2010-2013) - PCV13 (2010) | IPD/carriage | 3-67 years | 23B second most prevalent serotype (10%) 19A proportion halved to 5% compared to PCV10 era | 38% penicillin non-susceptible 56% co-trimoxazole non-susceptible |  |
| [30] | Portugal | 2015-2018 | PCV13 (2015) | IPD | ≥18 years | 23B enriched among CSF cases (n=5/113, *P*=0.002) 19A prevalence decreased to 5% (n=116) but still top-5 | top-4 NVT among penicillin non-susceptible isolates |  |
| [31] | Spain | 2004-2020 | PCV7 (2001) - PCV13 (2010) | IPD | >18 years | 23B cases increased post-PCV13 and now top-3 NVT 19A cases decreased and stabilized in past 5 years | increasing penicillin MIC90 during COVID-19 |  |
| [32] | Spain (Gipuzkoa) | 2008-2016 | PCV7 (2001) - PCV13 (2010) | otitis media | ≤14 years | 23B prevalence increased post-PCV13 and became top NVT (16% of all non-PCV13) 19A prevalence decreased post-PCV13 (29.2% to 3.8%, *P*=0.003) |  | all 23B ST2372 |
| [33] | Norway | 2004-2016 | PCV7 (2006) - PCV13 (2011) | IPD/carriage | all ages/1-5 years | 23B (mainly ST2372) caused peak in penicillin non-susceptible and co-trimoxazole resistant NVT IPD multi-drug resistant 19A IPD prevalence decreased post-PCV13 | third most common penicillin non-susceptible serotype (9%, n=28) fourth co-trimoxazole resistant serotype (7%, n=21) | ST2372 post PCV13 |
| [34] | USA | 1998-2013 | PCV7 (2000) - PCV13 (2010) | IPD | <5 years | penicillin non-susceptible 23B ST1373 peak in post-PCV13 IPD low prevalence of multi-drug resistant 19A post-PCV13 | penicillin non-susceptible 23B increase | ST1373 post-PCV13 |
| [35] | Germany | 1992-2014 | PCV7 (2006) - PCV10 (Feb 2009) - PCV13 (Dec 2009) | IPD | all ages | 23B prevalence increased significantly post-PCV13 (2007-2010: n=34, 0.5% to 2010-2014: n=265, 2.8%, *P<*0.001) | 46.5% penicillin non-susceptible of all 23B but increased post-PCV13 42.3% co-trimoxazole non-susceptible | ST439 and ST1349 most prevalent but also ST2372, ST9867, ST9872, and ST778 |
| [36] | Iceland | 2009-2017 | PCV10 (2012) | carriage | <7 years | 23B prevalence increased significantly post-PCV10 (from 2012-2017: n=1, 0.7/1,000 samples to 2009-2011: n =152, 49.3/1,000 samples; P<0.001) 19A prevalence remained stable (P=0.02) | often penicillin susceptible (n=120, 79%) | majority belongs to ST439 |
| [37] | Bulgaria | 1992-2013 | PCV10 (2010) | non-invasive isolates | ≤16 years | 19A most common NVT post-PCV10 (n=21, 13.8%) |  | ST1040, ST12066, ST189, ST2372* |
| [38] | Brazil | 2008-2013 | PCV10 (2010) | IPD | <5 years | Only one case of 23B IPD post-PCV10 19A most prevalent post-PCV10 (n=20, 13%) |  |  |
| [39] | Finland | 2010-2018 | PCV10 (2010) | IPD/carriage | 6-102 months | Only 6 cases of 23B IPD (4.6%) 19A most common NVT post-PCV10 (n=60, 46%) |  |  |
| *one single isolate | | | | | | | | |
